# Physics-Informed Neural-Network-Based Generation of Composite Representative Volume Elements with Non-Uniform Distribution and High-Volume Fractions

**DOI:** 10.3390/polym18010097

**Published:** 2025-12-29

**Authors:** Tianlu Zheng, Chaocan Cai, Fan Yang, Rongguo Wang, Wenbo Liu

**Affiliations:** National Key Laboratory of Science and Technology on Advanced Composites in Special Environments, Center for Composite Materials and Structures, Harbin Institute of Technology, Harbin 150080, China

**Keywords:** representative volume element, polymer composite materials, physics-informed neural network, non-uniform distribution, statistical analysis, micromechanical analysis, damage behavior analysis

## Abstract

To reduce the reliance on large training sets for representative volume element (RVE) generation using machine learning, this work presents a novel approach based on physics-informed neural network (PINN) to generate RVEs for unidirectional fiber-reinforced composites with non-uniform fiber distributions and high-volume fractions. The method embeds physical constraints including fiber non-overlap, volume fraction, and boundary conditions directly into the neural network’s loss function. This integration eliminates the need for large training datasets, which is typically required by traditional machine learning methods. Moreover, it achieves volume fractions exceeding 0.8, surpassing the jamming limit of conventional generation techniques. Exhaustive statistical measurements taken at different scales confirm that the proposed method could accurately reproduce local fiber distribution patterns in realistic microstructures while maintaining complete randomness at larger scales. Finite element analysis was employed on the generated RVEs to predict the elastic properties and damage behavior that taking into account the interfacial debonding and nonlinear damage in matrix. The predictions of both macroscopic mechanical properties (elastic properties and strength) and microscopic damage patterns show good agreement with experimental results. The proposed PINN-based framework provides an efficient and reliable tool for computational micromechanics of polymer matrix composites.

## 1. Introduction

The remarkable mechanical properties of fiber-reinforced composites—notably, their high specific strength and stiffness, low density, and versatile design options—have led to their widespread adoption in numerous industrial applications [1,2]. With the expanding market for these materials, there is an urgent need for methods that are both economical and accurate to predict their mechanical behavior. In recent years, micromechanics computation based on representative volume element (RVE) has been an efficient method to address this issue. Shahid et al. [3] developed three-dimensional RVE models for single-fiber, hybrid, and multi-fiber systems to predict their effective elastic properties. Zhang et al. [4] proposed an algorithm for generating RVE to mitigate microstructural inhomogeneities in fiber-reinforced composites. Ghayoor et al. [5] developed an algorithm to generate random RVE with varying volume fractions and minimum distances between fibers. In general, aerospace-grade high-strength fiber-reinforced composites feature a fiber volume fraction ranging from 60% to 70%, with the fibers being randomly distributed. This distribution nature plays a vital role in defining the overall mechanical behavior of fiber-reinforced composites, specifically influencing local stress transfer mechanisms and crack damage initiation phenomena (e.g., interfacial debonding, matrix cracking). Therefore, constructing an RVE model that accurately reflects the fiber distribution features in actual composite microstructures is key to overcoming the limitations of traditional macroscopic homogenized models, especially in predicting the mechanical behavior of heterogeneous materials and solving prediction challenges related to “structure-property-failure” relationships in composites [6,7,8].

To develop statistically equivalent representative volume elements of polymer-based composites, scholars have proposed various methods. The scanning–reconstruction method [9,10] involves obtaining the micro-images of the composites using SEM scanning and reconstructing the fiber distribution model via CAD. This method requires a significant amount of time and resources when micro-images with multiple fiber volume fractions are needed. Additionally, the random distribution model obtained through this method can only represent the fiber distribution in local regions. Therefore, effective algorithms and tools are needed to generate reinforcing phase random distribution morphologies in the matrix that reflect the real structural characteristics of composite materials. The hard-core algorithm, alternatively referred to as the random Sequential Adsorption (RSA) method, entails the sequential placement of fibers at random locations within a specified RVE until the target fiber volume fraction is achieved [11,12]. However, there is a jamming limit of about 0.54 on the fiber volume fraction, which limits the application of this method in creating RVE models with high fiber fraction in high-strength composite materials [13,14]. To generate RVEs with higher volume fractions, various approaches have been proposed. Some researchers have introduced improved algorithms based on the traditional RSA algorithm, such as the hardcore stirring model and the random sequential expansion algorithm [15,16,17]. Several experimentally numerical integrated methods for establishing statistically equivalent fiber distributions in composites have been developed, yet the experimental procedures still face dual challenges of cost and efficiency [18,19]. The initial periodic vibration models organize fibers into regular square or hexagonal patterns before introducing controlled disturbances to create RVEs containing randomly dispersed inclusions [20,21,22,23]. However, these methods fail to fully remove the inherent periodicity when fiber concentrations are high, leading to inadequate randomness in the produced RVEs. Molecular dynamics simulations efficiently generate RVEs with high volume fraction inclusions, but their numerical processing routines are extremely complex [24,25,26]. The displacement numerical optimization algorithm defines the degree of fiber overlap as a certain form of overlap potential energy and introduces methods to minimize it. The optimization algorithms used include the dynamic scheme incorporated with Box-2D [27], the maximum penetration-biased algorithm [6], L-BFGS-B optimization method [28], and gradient descent methods [29,30]. Displacement-based optimization methods present the capability to generate RVEs with fiber volume fractions above 0.8. Additionally, biomimetic-based optimization algorithms, such as the cuckoo search algorithm and the improved artificial bee colony algorithm, have also been used to generate RVEs with high fiber volume fractions [31,32,33]. Nevertheless, practical implementation of these algorithms usually involves some complexity. Overall, existing RVE generation methods primarily face three major challenges: high algorithmic complexity, difficulty achieving high volume fraction inclusion rates, and low computational efficiency. Recently, machine learning technology, such as deep learning and artificial neural networks, has shown impressive results and promising prospects in materials science research, with widespread applications in composite material microstructure identification, performance prediction, structural design, and damage detection [34,35,36,37]. However, the use of machine learning methods to generate RVEs with randomly distributed fibers is still rarely reported. Guo et al. [38] have developed a deep convolutional generative adversarial network to generate RVE models of composite materials, but it requires a large number of micro-images as training samples.

To avoid the large training dataset requirement in generating RVEs with machine learning technology, this work proposes a new, simple, and efficient algorithm based on physics-informed neural networks (PINN) to generate RVEs with non-uniform distribution and high fiber volume fractions. Section 2 elaborates on the proposed generation approach. Section 3 presents statistical validation through a comparison between generated RVEs and experimental measurements in realistic composite materials, as well as the elastic characteristics and damage mechanisms, considering interfacial debonding and nonlinear damage in the matrix of CFRPs subjected to transverse tensile and compressive loads. Finally, the concluding remarks are provided in Section 4.

## 2. RVE Generating Methods

**Figure 1:**
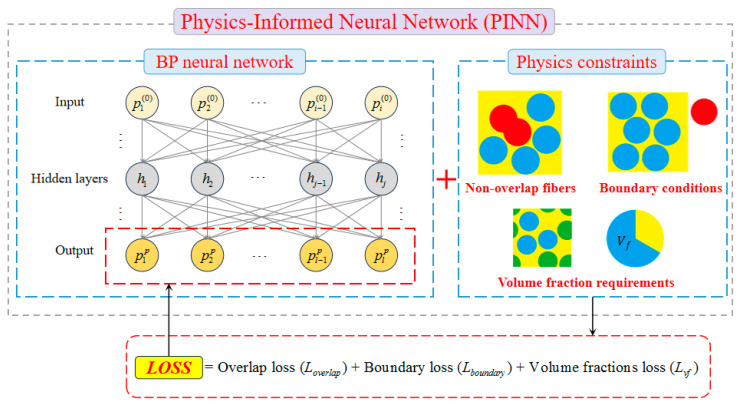
Framework of PINN-based method for generating RVE models.

Physics-informed neural networks (PINN) comprise an emerging methodology that embeds physical laws directly into neural architectures in machine learning [39,40,41]. The foundational principle involves modifying the original loss function by incorporating physical principles as constraints, aiming to minimize the weights and biases of the neural network to ensure that computations adhere to fundamental physical laws during model optimization. The proposed PINN-based approach for generating RVEs with high fiber volume fractions and random fiber distributions is illustrated in Figure 1, with its implementation details presented in the following section.

### 2.1. Initialization of Fiber Coordinates

The generation of the RVE with randomly distributed fibers is achieved by adjusting fibers that appear randomly in space to satisfy the physical rule of non-overlapping in space, thus requiring the generation of a series of randomly distributed fibers as initialization parameters. In this work, the initial position $p_{i}^{\left(0\right)}=(x_{i}^{\left(0\right)},\;y_{i}^{\left(0\right)})$ of the fibers is randomly generated within the polar axis interval $\left[-\frac{L}{2},\;\frac{L}{2}\right]$, following a uniform spatial distribution:

(1) $$p_{i}^{\left(0\right)}=(x_{i}^{\left(0\right)},\;y_{i}^{\left(0\right)})\sim U{\left[r_{f},\;L-r_{f}\right]}^{2}$$

where $r_{f}$ is the fiber radius and *L* is the RVE edge length dynamically calculated based on the target volume fraction $V_{f}$:

(2) $$L=\sqrt{\frac{N\pi r_{f}^{2}}{V_{f}}}$$

where *N* represents the total number of fibers and $V_{f}$ is the fiber volume fraction.

### 2.2. Definition of Loss Function

The loss function of the PINN-based generation method is constructed by considering three physical properties of the RVE: spatial non-overlapping of fibers, required fiber volume fraction, and partial inclusion of each fiber within the RVE boundary.

Given the non-overlapping spatial nature of fibers, a loss function $L_{overlap}$ for radius overlap measurement between different fibers is defined as

(3) Loverlap=∑i=1N∑j≠iNReLU(ri+rj+dmin−dij)

where $r_{i}$ and $r_{j}$ are the radius of fiber i and j, respectively; $d_{ij}$ indicates the distance between fiber i and j; and $d_{\min}$ is the minimum distance required in fibers. The ReLU function, as shown in Equation (4), serves to compare fiber minimum effective spacing against the required distance and assess optimization completion status. A linear penalty function will be introduced to guide fiber repositioning and avoid overlap if $d_{ij}\geq r_{i}+r_{j}+d_{min}$.

(4) ReLU(x)=max(0,x)

Because fibers must be entirely contained within the RVE domain, a loss function needs to be established to verify whether fiber positions comply with the spatial constraints of the RVE. The loss function $L_{boundary}$, defined by Equation (5), identifies out-of-bound fibers and imposes penalties, thereby ensuring fibers are fully contained within the RVE region.

(5) $$L_{boundary}=\sum_{i=1}^{N}\left[\mathrm{ReLU}(-x_{i})+\mathrm{ReLU}(x_{i}-L)+\mathrm{ReLU}(-y_{i})+\mathrm{ReLU}(y_{i}-L)\right]$$

Additionally, to maintain the initial fiber volume fraction of the RVE and generate a statistically representative periodic microstructure, it is necessary to add replica fibers at corresponding positions where fibers intersect the boundaries to construct the periodic microstructure. And, the loss function is defined as

(6) Lvf=∑i=1Nc∑j≠iNaReLU(ri+rj+dmin−dij)

where $N^{c}$ is the number of fibers overlapping with boundaries and B denotes the total fiber count, including both initial fibers and replicated fibers.

Weighing each loss component and combining them through linear summation, the developed PINN framework can produce RVEs featuring randomly distributed fibers. Therefore, the total loss function is defined as follows:

(7) $$L_{total}=\alpha_{o}L_{overlap}+\alpha_{b}L_{boundary}+\alpha_{vf}L_{vf}$$

In this work, $\alpha_{o}$, $\alpha_{b}$, and $\alpha_{vf}$ take the value of 1000.

### 2.3. Backpropagation Neural Network and Optimization Process

A backpropagation neural network is used for training in this study, with its structure consisting of an input layer, two hidden layers, and an output layer. Each hidden layer comprises 128 neurons, with full connections between neurons at all levels. The information flow of the PINN model based on the BP neural network is controlled by the following equation:

(8) $$\left\{\begin{array}{l} h_{j}^{I}=\tanh(\sum_{i=1}^{2N}w_{i}^{\left(h_{j}^{I}\right)}p_{i}^{\left(0\right)}+b_{j}^{I})\\ h_{k}^{II}=\tanh(\sum_{j=1}^{128}w_{j}^{\left(h_{k}^{II}\right)}h_{j}^{I}+b_{k}^{II})\\ p_{l}^{p}=\sum_{k}^{128}w_{k}^{\left(p_{i}^{p}\right)}h_{k}^{II}+b_{l}^{p} \end{array}\right.$$

where $h_{j}^{I}$ and $h_{k}^{II}$ are the neurons in hidden layer 1 and hidden layer 2, respectively; w is the weight parameter of each neuron, where the superscript represents the corresponding neuron and the subscript corresponds to the number of the previous-level neuron connected to this neuron; and b is the bias for each neuron, with the subscript corresponding to the respective neuron.

The adjustment direction of the weight parameters (*w*) and bias (*b*) of each layer in the neural network is determined by the gradient of the total loss $L_{total}$:

(9) $$\left\{\begin{array}{l} \nabla_{W^{p}}L=\sum_{l=1}^{2N}\sum_{k=1}^{128}h_{k}^{II}\frac{\partial L}{\partial p_{l}^{p}}\\ \nabla_{W^{II}}L=\sum_{l=1}^{2N}\sum_{k=1}^{128}\sum_{j=1}^{128}h_{j}^{I}{\left(t_{k}^{II}\right)}^{2}w_{k}^{\left(p_{l}^{p}\right)}\frac{\partial L}{\partial p_{l}^{p}}\\ \nabla_{W^{I}}L=\sum_{l=1}^{2N}\sum_{k=1}^{128}\sum_{j=1}^{128}\sum_{i=1}^{2N}p_{i}^{\left(0\right)}t_{j}^{I}w_{j}^{\left(h_{k}^{II}\right)}{\left(t_{k}^{II}\right)}^{2}w_{k}^{\left(p_{l}^{p}\right)}\frac{\partial L}{\partial p_{l}^{p}} \end{array}\right.$$

(10) $$\left\{\begin{array}{l} \nabla_{b^{p}}L=\sum_{i=1}^{2N}\frac{\partial L}{\partial p_{i}^{p}}\\ \nabla_{b^{II}}L=\sum_{l}^{2N}\sum_{k=1}^{128}{\left(t_{k}^{II}\right)}^{2}w_{k}^{\left(p_{l}^{p}\right)}\frac{\partial L}{\partial p_{l}^{p}}\\ \nabla_{b^{I}}L=\sum_{l=1}^{2N}\sum_{k=1}^{128}\sum_{j=1}^{128}t_{j}^{I}w_{j}^{\left(h_{k}^{II}\right)}{\left(t_{k}^{II}\right)}^{2}w_{k}^{\left(p_{l}^{p}\right)}\frac{\partial L}{\partial p_{l}^{p}} \end{array}\right.$$

where

(11) $$\left\{\begin{array}{c} t_{j}^{I}=1-\tanh^{2}(w_{i}^{\left(h_{j}^{I}\right)}p_{i}^{\left(0\right)}+b_{j}^{I})\\ t_{k}^{II}=1-\tanh^{2}(w_{j}^{\left(h_{k}^{II}\right)}h_{j}^{I}+b_{k}^{II}) \end{array}\right.$$

In each iteration, the adjustment direction of w and b is opposite to the gradient mentioned above, and the adjustment length is *α*, called the learning rate, as follows:

(12) $$\left\{\begin{array}{l} w_{t+1}=w_{t}+\alpha_{w}{\left(\nabla_{W}L\right)}_{t}\\ b_{t+1}=b_{t}+\alpha_{b}{\left(\nabla_{b}L\right)}_{t} \end{array}\right.$$

where *t* is the t-th iteration step and $\alpha_{w}$ and $\alpha_{b}$ are the learning rate for the weight parameter and bias. To improve convergence, the adaptive moment estimation (Adam) optimizer is introduced for the iterative optimization process [42].

## 3. Results and Discussion

### 3.1. RVE Generation

**Figure 2:**
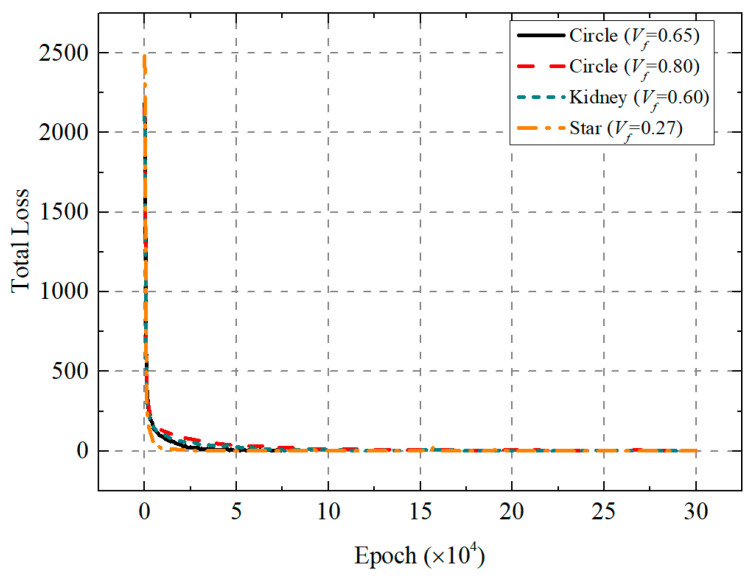
Total loss of generating RVEs with different fiber shapes.

**Figure 3:**
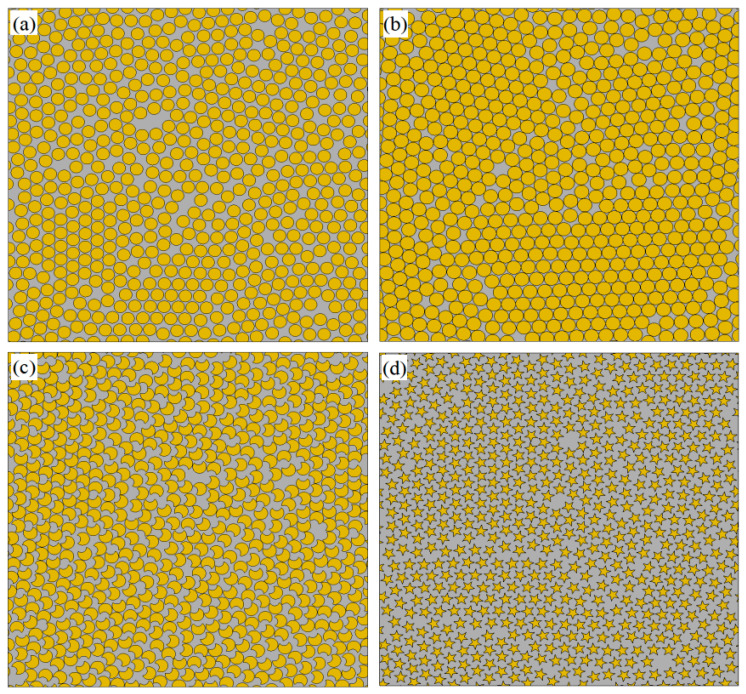
RVEs generated by the PINN-based method. (**a**) Circle with $V_{f}=0.65$. (**b**) Circle with $V_{f}=0.80$. (**c**) Kidney with $V_{f}=0.60$. (**d**) Star with $V_{f}=0.27$.

Figure 2 presents the evolution curves of the total loss function when the PINN generates RVEs with different fiber shapes. It is observed that the loss functions for all cases decrease sharply during the initial training phase and then converge toward zero with oscillations. This phenomenon demonstrates the ability of the PINN-based method to accommodate varying volume fraction requirements while achieving stable convergence, underscoring the reliability of this method in RVE generation. The final geometries of the generated RVEs with circle fibers are shown in Figure 3a,b. The maximum achievable volume fraction is higher than that reported in [9,10,11,12,13,14,18]. Notably, as shown in Figure 3c,d, this method can also generate RVEs with non-circular fiber cross-sections and different volume fractions by constructing a circumcircle. Table 1 summarizes the performance of different RVE generation methods in terms of algorithmic complexity, maximum achievable volume fraction, and computational efficiency. In the case of $V_{f}$ = 0.65, the time for generating the RVE with uniform radius circular fibers (Nf = 518) by the proposed algorithm is about 1.35 min. For comparison, the generation times of Melro et al. [15], Wongsto et al. [21], Cai et al. [31], and Pathan et al. [28] on a computer with specifications similar to the one used in this work are 3.31 min, 7.20 min, 26.08 min, and 107.02 min, respectively.

### 3.2. Statistical Characterization Analysis

**Figure 4:**
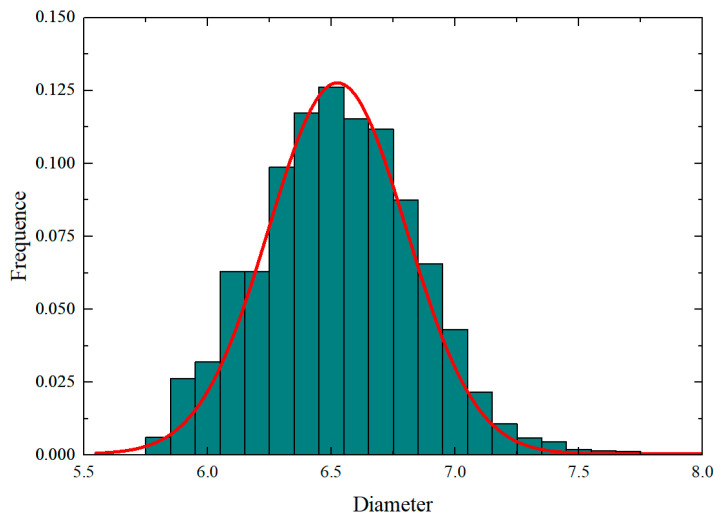
Size distribution of fibers.

To assess the effectiveness of the proposed PINN algorithm in generating RVEs, statistical methods were applied to analyze the fiber spatial arrangement [18,43]. The foremost aim was to examine the statistical randomness of fiber distributions to ensure they match actual composite microstructures. This research utilized three statistical metrics: nearest neighbor distances (NND), second-order intensity function, and pair distribution function. A total of 100 RVE samples were generated with a fiber volume fraction of 0.6, featuring normally distributed fiber diameters averaging 6.6 μm with a 0.3106 standard deviation, the size distribution of fibers adapted from Ref. [18] are presented in Figure 4.

#### 3.2.1. Nearest Neighbor Distances

**Figure 5:**
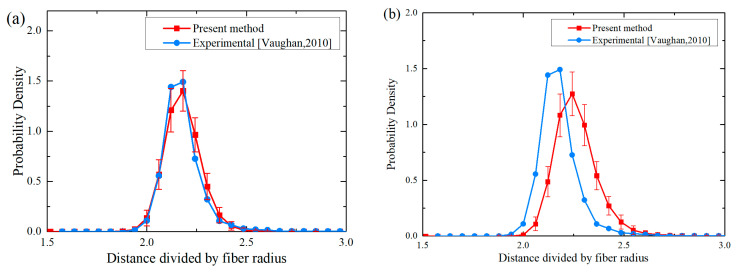
Comparison of first (**a**) and second (**b**) nearest neighbor distances with experimental data from Ref. [18].

The nearest neighbor distance is a fundamental function for statistically characterizing spatial interaction point systems. These probability density functions represent the random distance from a reference fiber to its nearest neighbor, which provides insight into the distribution of the generated fibers. Figure 5a,b compare the probability density functions of the first and second nearest neighbor distances of the generated RVEs with the results obtained from the experiment. Error bars are included to show the range of fluctuations for each data point. It can be noted that the statistical results from the proposed PINN-based method show high consistency with the observed experimental data [18]. These research outcomes substantiate that the proposed algorithm generates fiber distribution patterns of high fidelity to real composites at the crucial short-range scale for fiber interactions.

#### 3.2.2. Second-Order Intensity Function

The second-order intensity function (also known as Ripley’s K function) is another widely used statistical metric for analyzing the spatial distribution of fibers. This function not only provides the local fiber distribution information but also effectively reflects the long-range interactions between fibers. It is defined as the ratio of the number of additional fibers within a circular area centered on any fiber with a radius of r to the number of fibers per unit area. Mathematically, it is defined as follows:

(13) $$K(r)=\frac{V}{N^{2}}\sum_{i}\sum_{j\neq i}\frac{I(d_{ij}\leq r)}{w(i,j)}$$

where *V* is the volume of the RVE, *N* is the total number of fibers within the RVE, *I*(·) is the indicator function, which takes the value of 1 when the expression in parentheses is true and 0 otherwise, $d_{ij}$ represents the distance from fiber *i* to fiber *j*, and *w*(*i*, *j*) is the weight function, which takes the value of 1 when the circle centered on fiber *i* and passing through fiber *j* lies entirely within the RVE; otherwise, it takes the ratio of the arc length of the circle within the RVE to the circumference.

For the CSR pattern, the theoretical K(r) is defined by Equation (14). When the plot of K(r) for a given fiber distribution lies above the CSR curve, it suggests some degree of clustering, while positions below demonstrate periodic patterns.

(14) $$K_{CSR}(r)=\pi r^{2}$$

**Figure 6:**
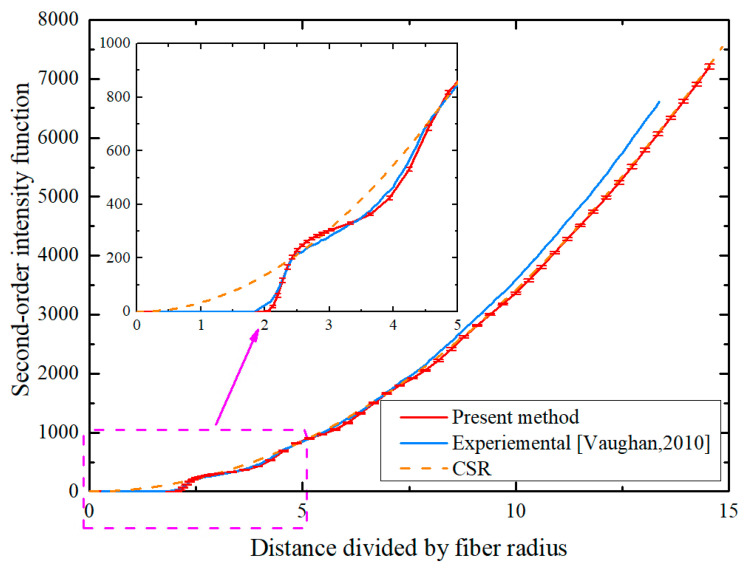
Comparison of second-order intensity function with CSR pattern and experimental data from Ref. [18].

Figure 6 presents the second-order intensity function of RVE generated by the PINN algorithm, with experimental data and the CSR pattern provided as reference benchmarks. It can be found that at short distances, the experimental data exhibit a distinct step-like curve shape and the proposed algorithm’s K(r) curve shows excellent agreement with the experimental result [18]. This suggests that the proposed PINN algorithm could accurately reconstruct the fiber spatial distribution in real composite materials. With the distance increasing, the experimental data begin to deviate from the CSR model. However, the proposed PINN algorithm remains highly consistent with the CSR pattern, consistently maintaining random distribution characteristics.

#### 3.2.3. Pair Distribution Function

The pair distribution function, alternatively named the radial distribution function (RDF), characterizes the probability distribution for fiber’s presence in a ring-shaped domain with inner radius r and radial thickness dr. It offers crucial insights into spatial patterning that work in tandem with Ripley’s functional analysis. Its mathematical definition is given by

(15) $$g(r)=\frac{1}{2\pi r}\cdot \frac{dK(r)}{dr}$$

**Figure 7:**
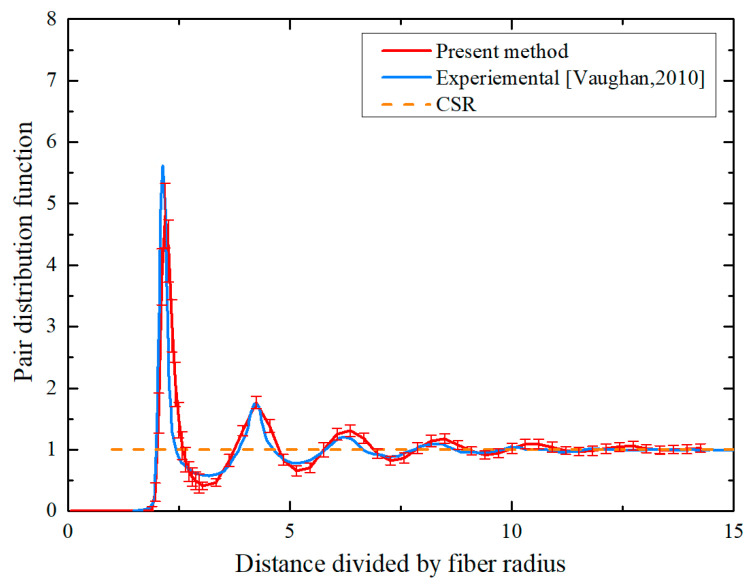
Comparison of pair distribution function with CSR pattern and experimental data from Ref. [18].

When Equation (15) is substituted into g(r) in place of K(r), the g(r) of the CSR model becomes identically 1 at every distance. Figure 7 illustrates the g(r) function acquired from the RVEs created using the presented PINN method, with experimental data and the CSR model included for comparison. It is observable in the figure that a pronounced peak emerges in the experimental data curve at around $2r_{f}$ distance, subsequently decaying in an oscillatory manner as distance increases. The peak produced by the proposed method aligns closely with the experimental data in both amplitude and location [18], reaffirming its ability to accurately replicate the fiber distribution patterns at short distances in real composite structures. Oscillatory convergence towards unity is observed for both curves with increasing distance, signaling fiber distributions approaching complete spatial randomness. These results demonstrate that the proposed algorithm successfully generates fiber distribution patterns closely resembling those in actual composites again, especially at the critical short-range scale where local fiber interactions dominate. It is confirmed that the PINN algorithm proposed in this paper provides a robust and efficient instrument for micromechanical analysis in composite materials.

### 3.3. Mechanical Performance Prediction

Accurate prediction of macroscopic mechanical characteristics for composites based on constituent material properties is the foremost objective of microstructure generation algorithms. It serves as the direct measure of their validity. This study establishes a finite element analysis-based verification framework to assess the reliability of PINN-generated RVEs for mechanical property predictions. This framework covers both effective elastic properties and damage mechanisms.

#### 3.3.1. Effective Elastic Property Prediction

The effective elastic properties of composites were predicted using RVEs generated by the proposed PINN-based method. Periodic boundary conditions were imposed on each corresponding node pair along the opposing edges of RVEs. The effective elastic properties were derived using the following volume homogenization equation [44,45]:

(16) $$E_{j}=\frac{\sum_{i=1}^{N^{e}}\sigma_{jj}^{i}A^{i}}{\sum_{i=1}^{N^{e}}\varepsilon_{jj}^{i}A^{i}}\;\upsilon_{jk}=-\frac{\sum_{i=1}^{N^{e}}\varepsilon_{kk}^{i}A^{i}}{\sum_{i=1}^{N^{e}}\varepsilon_{jj}^{i}A^{i}}\;G_{jk}=\frac{\sum_{i=1}^{N^{e}}\sigma_{jk}^{i}A^{i}}{\sum_{i=1}^{N^{e}}\varepsilon_{jk}^{i}A^{i}}$$

where $N^{e}$ is the total number in RVE, $\sigma_{jj}^{i}$ and $\varepsilon_{jj}^{i}$ correspond to the stress and strain values in the j-direction of element i, and $A^{i}$ represents the area of the element i.

In this section, the composite material E-glass/MY750/HY917/DY063 was employed, featuring isotropic behavior in both the fiber and the matrix with parameters of $E_{f}$ = 74 Gpa, $\upsilon_{f}$ = 0.2, $E_{m}$ = 3.35 GPa, and $\upsilon_{m}$ = 0.35 [46]. The finite element models were constructed using Abaqus/standard software integrated with Python (version 3.9) scripts, primarily utilizing CPE4 elements, along with some CPE3 elements. Twenty RVEs featuring random fiber distributions at $V_{f}$ = 0.6 were created, each comprising 100 fibers and having a mesh element size of about 0.1 $r_{f}$. The elastic properties derived from the simulation are displayed in Table 2, along with computational results from alternative algorithms [15,16] and experimental data [46]. It can be observed that the results obtained from the RVEs generated by the PINN-based method proposed in this paper are acceptable for the experimental data and outperform those of other algorithms. It is worth mentioning that the predicted transverse elastic and shear modulus are somewhat smaller than the experimental data. This can be attributed to the interphases present in the actual composites, which were not considered in the numerical simulations [16]. Ge et al. [47] indicated that the modulus of the interphases has a significant impact on the transverse elastic modulus and shear modulus while having almost no effect on the Poisson’s ratio of the composite material. And, as shown in Table 2, the predicted Poisson’s ratio aligns well with the experimental results, providing support for the numerical predictions. Additionally, the data in Table 3, showing values nearly equal to 1.0, confirm that the microstructure produced by the presented algorithm exhibits transverse isotropy, as anticipated for fiber-reinforced composites.

Additionally, this section examines the carbon fiber composite material HTA/6376 [48] and the constituent properties are listed in Table 4. 3D RVEs were developed to predict the elastic properties of the composites. The same meshing strategy used in Ref. [31], primarily using C3D8R elements with a small proportion of C3D6 elements, was applied in this study. Each model contains approximately 160,000 elements, and mesh convergence has been verified. Periodic boundary conditions were adopted in the simulations. The average effective elastic properties obtained from twenty different RVEs are summarized in Table 5. It is evident that the predicted results correlate well with the experimental data [49]. Thus, the RVE generation method proposed in this work can be reliably employed to predict the elastic properties of composites.

#### 3.3.2. Strength and Damage Behavior Analysis

To study the damage behavior of composite materials, the effects of hydrostatic stress and damaged plastic deformation in the polymer matrix are taken into account in this section, with the implementation of the extended linear Drucker–Prager yield criterion shown in Equation (17) [50,51].

(17) $$\frac{1}{2}q\left[1+\frac{1}{k}-(1-\frac{1}{k}){\left(\frac{r}{q}\right)}^{3}\right]-p\tan\beta -d=0$$

where p and q correspond to the hydrostatic pressure and Mises stress, β is the friction angle, *d* denotes the matrix cohesion, *r* indicates the third invariant of deviatoric stress, and *k* represents the ratio of triaxial tensile yield stress to triaxial compressive yield stress.

**Figure 8:**
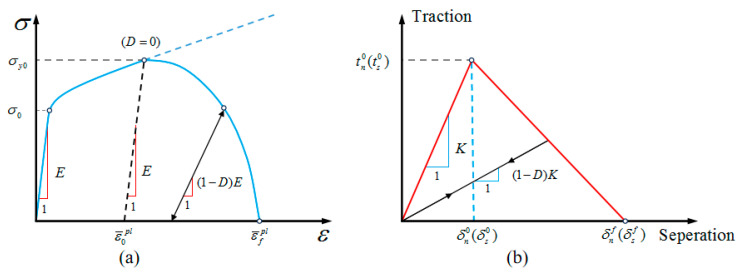
The stress–strain response curve of the matrix (**a**) and the interface (**b**).

Experimental investigations reveal that polymers demonstrate a brittle fracture at low strain when subjected to uniaxial tension but yield and undergo significant plastic deformation under uniaxial compression [52,53]. Therefore, a ductility criterion is introduced in this work to capture this behavior, where the initiation of damage under differing loading conditions is assessed using the stress triaxiality η=−p/q. For simplification, the epoxy resin is assumed to be an ideal plastic material in this study, with η values of 1/3 under transverse tension and −1/3 under transverse compression. Following damage initiation, the development of damage within the matrix is dictated by a progressive failure process involving very little fracture energy. The stress–strain response of this process is shown in Figure 8a.

Given the critical influence of the fiber/matrix interface on composite damage behavior, this study employed zero-thickness cohesive elements to model interface debonding. As shown in Figure 8b, the constitutive behavior of the cohesive element is described by a bi-linear traction–separation curve, where the initial elastic portion is controlled by stiffness parameter K [51]:

(18) $$\left\{\begin{array}{l} t_{n}=K\delta_{n}\\ t_{s}=K\delta_{s} \end{array}\right.$$

The initiation of damage is considered to happen when the maximum stress criterion is satisfied:

(19) $$\max\left\{\frac{\langle t_{n}\rangle }{t_{n}^{0}},\frac{t_{s}}{t_{s}^{0}}\right\}=1$$

where 〈〉 denotes Macaulay brackets and represents the normal and tangential interface strengths, respectively.

Additionally, experimental findings indicate that fiber breakage typically does not occur under transverse loading. Therefore, fibers are modeled as undamaged linear elastic isotropic materials. The material parameters used in this section are detailed in Table 6 [54].

**Figure 9:**
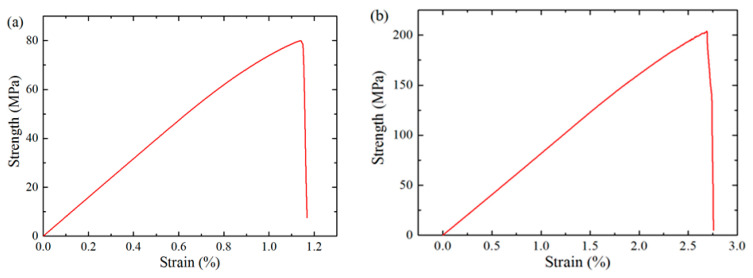
Stress–strain responses of RVE under transverse tension (**a**) and compression (**b**).

**Figure 10:**
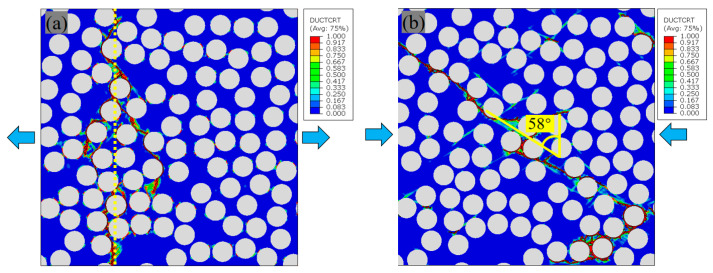
Damage patterns of RVE under transverse tensile (**a**) and compressive (**b**).

Figure 9 presents the mechanical response of PINN-generated RVE under tension and compression, where the numerically obtained transverse elastic modulus (8.02 GPa), tensile strength (79.98 MPa), and compressive strength (203.95 MPa) demonstrate satisfactory consistency with the experimental data (7.74 GPa, 76.8 MPa, 189.7 MPa) [54]. The relative deviations are 3.62%, 4.14%, and 7.51%, respectively. When subjected to tensile load, initial interfacial damage and matrix plasticity occur in regions of minimum fiber spacing, ultimately resulting in a primary damage band oriented perpendicular to the load direction, as shown in Figure 10a. This result is in accordance with the experimental observations in Ref. [54].

On the other hand, when a solid follows the Mohr–Coulomb criterion and undergoes uniaxial compression, its fracture surface inclines at an angle of $\alpha =45{}^{\circ}+\frac{\varphi }{2}$ relative to the plane perpendicular to the loading axis [2]. In this work, the value of φ=26° gives α=58°, and the damage morphology under transverse compression reveals that the plastic shear band forms an angle of α with the loading direction’s perpendicular plane, measuring approximately 58°, illustrated in Figure 10b, which aligns with the theoretical value and the experimental observation in Ref. [54]. These results demonstrate that the PINN-based approach serves as an efficient method for investigating micromechanical damage in composite materials.

## 4. Conclusions

This paper proposes a novel method for generating representative volume elements (RVEs) with non-uniform fiber distributions and high-volume fractions for composite materials using physics-informed neural networks (PINNs). The key findings of this study can be summarized as follows:

1. The PINN-based method proposed in this work eliminates the reliance on massive training sets required by conventional neural networks and overcomes the jamming limit in traditional generation techniques like RSA, raising the maximum achievable volume fraction to 0.8 while simultaneously enabling controllable spatial gaps in fiber arrangements.
2. Statistical examinations involve nearest neighbor distances, the second-order intensity function, and the pair distribution function conducted on the generated RVEs. These examinations reveal that the PINN-based methodology can accurately reconstruct fiber spatial distributions observed in actual composite materials, particularly at the crucial short-range level where fiber interactions are most significant.
3. Finite element simulations were conducted on RVEs generated by the proposed method to predict their elastic properties and damage behaviors. The results show that the predictions are consistent with experimental data, validating the effectiveness of the PINN-based method in generating RVEs for micromechanical studies in composite materials.

## Figures and Tables

**Table 1:** Comparison of RVE generation methods.

Method	Algorithm Complexity	Achievable Maximum Volume Fraction	Computational Efficiency
Scanning–reconstruction method	Easy to implement, requires substantial resources	Depends on the actual microstructures	Low
RSA algorithms	Easy to implement	<0.54	Depends on volume fraction
Modified RSA algorithms	Depends on the algorithm	Depends on the algorithm	Depends on the algorithm
Initial periodic vibration models	Complex	Depends on the algorithm, cannot eliminate the initial pattern in high volume fraction	Depends on the algorithm
MD-based method	Complex	>0.8	High
Displacement-based optimization method	Depends on the algorithm	>0.8	Depends on optimization method
Biomimetic-based optimization method	Complex	Depends on the algorithm	Depends on optimization method
General machine learning method	Fair, requires substantial extensive training data	>0.8	Low, training data preparation requires time
PINN-based method (presented work)	Easy to implement, no training samples required	>0.8	High

**Table 2:** Elastic properties obtained from calculations of generated RVEs.

	$E_{2}$ (GPa)	$\upsilon_{23}$	$E_{3}$ (GPa)	$\upsilon_{32}$	$G_{23}$ (GPa)
Average	13.935	0.389	13.924	0.392	4.657
Melro et al. [15]	13.376	0.370	13.387	0.371	4.851
Yang et al. [16]	13.047	0.405	13.068	0.405	4.673
Experiment [46]	16.2	0.4	16.2	0.4	5.786
Error (%)	13.98	2.75	14.05	2.00	19.51

**Table 3:** Transverse isotropy relationships of elastic properties.

	$\frac{E_{2}\upsilon_{32}}{E_{3}\upsilon_{23}}$	$\frac{E_{2}}{E_{3}}$	$\frac{\upsilon_{32}}{\upsilon_{23}}$	$\frac{E_{2}}{2(1+\upsilon_{23})G_{23}}$
Average	1.01	1.00	1.01	1.08

**Table 4:** The constituent properties of HTA/6376 composites.

Fiber (HTA)	$E_{11}$	$E_{22}=E_{33}$	$G_{12}=G_{31}$	$G_{23}$	$\nu_{12}$	$\nu_{23}$	$\nu_{31}$
	238 GPa	28 GPa	24 GPa	7.2 GPa	0.25	0.33	0.02
Matrix (6376)	E=3.63 GPa, ν=0.34						

**Table 5:** Elastic properties of HTA/6376 composites.

Elastic Properties	$E_{11}$ (GPa)	$E_{22}$ (GPa)	$E_{33}$ (GPa)	$G_{12}$ (GPa)	$\nu_{12}$
Experimental [49]	139	10	10	5.2	0.32
Average values	142.32	10.32	10.24	4.96	0.29
Errors (%)	2.39	3.20	2.40	4.62	9.38

**Table 6:** Mechanical properties of fiber, matrix, and interphase.

Fiber	$E_{f}$ (GPa)	$\upsilon_{f}$			
	23.34	0.25			
Matrix	$E_{m}$ (GPa)	$\upsilon_{m}$	$\sigma_{t}$ (MPa)	$\sigma_{c}$ (MPa)	*d* (MPa)
	3.45	0.35	85.7	232.5	104.8
	*β*	*k*	${\bar{\varepsilon }}_{0+}^{pl}$	${\bar{\varepsilon }}_{0-}^{pl}$	$G_{m}$ (J/m^2^)
	37.7°	0.8	0.025	0.25	5
Interphase	$K_{n}$$\;=\;K_{s}$ (GPa/m)	$t_{n}^{0}$$\;=\;t_{s}^{0}$ (MPa)		$G_{n}=G_{s}$ (J/m^2^)	
	$10^{8}$	85.7		100	

## Data Availability

The raw data supporting the conclusions of this article will be made available by the authors on request.
